# Partial Dominance, Overdominance and Epistasis as the Genetic Basis of Heterosis in Upland Cotton (*Gossypium hirsutum* L.)

**DOI:** 10.1371/journal.pone.0143548

**Published:** 2015-11-30

**Authors:** Qingzhi Liang, Lianguang Shang, Yumei Wang, Jinping Hua

**Affiliations:** 1 Department of Plant Genetics and Breeding/Key Laboratory of Crop Heterosis and Utilization of Ministry of Education, China Agricultural University, Beijing, 100193, P. R. China; 2 Research Institute of Cash Crops, Hubei Academy of Agricultural Sciences, Wuhan, 430064, Hubei, China; USDA-ARS-SRRC, UNITED STATES

## Abstract

Determination of genetic basis of heterosis may promote hybrid production in Upland cotton (*Gossypium hirsutum* L.). This study was designed to explore the genetic mechanism of heterosis for yield and yield components in F_2: 3_ and F_2: 4_ populations derived from a hybrid ‘Xinza No. 1’. Replicated yield field trials of the progenies were conducted in 2008 and 2009. Phenotypic data analyses indicated overdominance in F_1_ for yield and yield components. Additive and dominance effects at single-locus level and digenic epistatic interactions at two-locus level were analyzed by 421 marker loci spanning 3814 cM of the genome. A total of 38 and 49 QTLs controlling yield and yield components were identified in F_2: 3_ and F_2: 4_ populations, respectively. Analyses of these QTLs indicated that the effects of partial dominance and overdominance contributed to heterosis in Upland cotton simultaneously. Most of the QTLs showed partial dominance whereas 13 QTLs showing overdominance in F_2:3_ population, and 19 QTLs showed overdominance in F_2:4_. Among them, 21 QTLs were common in both F_2: 3_ and F_2: 4_ populations. A large number of two-locus interactions for yield and yield components were detected in both generations. AA (additive × additive) epistasis accounted for majority portion of epistatic effects. Thirty three complementary two-locus homozygotes (11/22 and 22/11) were the best genotypes for AA interactions in terms of bolls per plant. Genotypes of double homozygotes, 11/22, 22/11 and 22/22, performed best for AD/DA interactions, while genotype of 11/12 performed best for DD interactions. These results indicated that (1) partial dominance and overdominance effects at single-locus level and (2) epistasis at two-locus level elucidated the genetic basis of heterosis in Upland cotton.

## Introduction

Cotton (*Gossypium* spp.) is the most important fiber crop in the world. A number of experiments showed that significant heterosis existed in Upland cotton for yield and yield components [1–3]. Hybrids with acceptable heterosis have been released for cotton production in both India, and China,.

Rapid advances in high density molecular genetic linkage maps have enabled a fine dissection of quantitative traits into genetic effects of individual Mendelian loci [4]. To understand the genome of cotton and detect quantitative trait loci (QTLs) for lint yield and fiber quality, the first genetic linkage map was constructed in cotton [5]. Since then, a variety of genetic linkage maps with improved high density were constructed in different mapping populations derived from either interspecific tetraploids or cultivated allotetraploid cotton [6–12]. Because genetic polymorphism in intraspecific Upland cotton was lower than that in interspecific tetraploids, the genome coverage of genetic linkage maps in intraspecific tetraploids was relatively low [13]. An intraspecific genetic linkage map comprising 604 loci and covering 3141 cM was recently constructed in Upland cotton [14]. Some other Upland cotton intraspecific linkage maps were also reported such as a linkage map with 471 loci and coverage of 3070 cM [15], and another linkage map with 978 loci and coverage of 4,184 cM [16]. More recently, a genetic map was constructed containing 421 SSR loci with a span of 3814.3 cM using an F_2_ population in Upland cotton [17]. Moreover, the most marker-rich intraspecific linkage map, which contained 1540 loci and spanned 2,842.06 cM, was constructed using recombinant inbred line (RIL) population derived from a cross between Upland cotton cultivar/line ‘Yumian 1’ and ‘7235’ [18]. QTL mapping of yield and fiber quality traits based on high-density genetic linkage maps of intraspecific tetraploids can be very useful in revealing the genetic basis of lint yield and fiber properties. Recently, Liu et al. (2012) identified a total of 225 QTLs controlling yield and its components in both the XZM2-derived RILs and IF_2_ populations, and from a total of 111 non-redundant QTLs, of these, 23 were detected in both two populations simultaneously [19]. Shang et al. (2015) mapped a total of 20 QTLs for fiber quality traits using 177 RILs derived from ‘Xinza No. 1’, a cross between ‘GX11359’ and ‘GX100-2’ [20]. Sequences of the D5-genome of *G*. *raimondii*, A2-genome of the *G*. *arboreum* and genome sequence of cultivated Upland cotton (*Gossypium hirsutum* TM-1) were publically available recently [21–25]. These physical maps provide new opportunities to develop rich SSR and functional markers for construction of high-density genetic maps and further dissect the genetic basis of complex quantitative traits as well as heterosis in Upland cotton.

Utilizations of heterosis have made significant economic benefits in crops during the last century. However, the mechanism of heterosis remains enigmatic [26]. Different hypotheses including dominance hypothesis [27–29], overdominance hypothesis [30–31] and epistasis hypothesis [32–34] were proposed to explore the mechanisms of heterosis. Epistasis is a phenomenon which the effect of one gene is modified by one or several other genes. Epistasis can be contrasted to dominance, which is an interaction between alleles at the same gene locus. The dominance hypothesis attributes the superiority of hybrids to the suppression of undesirable (deleterious) recessive alleles from one parent by dominant alleles from another parent. The overdominance hypothesis states that some combinations of alleles are especially advantageous when paired in a heterozygous individual. Recent research using quantitative molecular tools provided new evidence for different hypotheses in dissecting heterosis [26, 35–38]. In two rice BC_1_F_7_ populations between recombinant inbred lines (RIL) and their parents, the heterozygotes were superior to the respective homozygotes in most QTLs, which supported the hypothesis that dominance was the genetic basis of heterosis in hybrid rice [39]. Stuber et al. [40] studied heterosis in the elite maize hybrid ‘B73 × Mo17’ and concluded that overdominance (or pseudo-overdominance) (*i*.*e*., a higher yield in the heterozygote than that in either homozygote) was the major cause of heterosis for grain yield. The hypothesis for epistasis as the genetic basis of heterosis was supported by a series of researches too [41–43]. The fact that a large number of digenic interactions for yield and yield components were detected in an F_2: 3_ population provided evidence for epistasis as the primary genetic basis of heterosis in rice [43]. Hua et al. [41–42] and Zhou et al. [44] further verified that heterozygotes were not necessarily advantageous for phenotypic traits and epistasis was the important genetic basis of heterosis in elite rice hybrid ‘Shanyou 63’. Multiple genetic mechanisms were identified to play roles in heterosis. In rapeseed, epistasis as well as all levels of dominance from partial to overdominance were responsible for the expression of heterosis [45]. Another study in hybrid maize ‘Yuyu No. 22’ suggested that genetic basis of grain yield heterosis relied on the cumulative effects of dominance, overdominance, and epistasis by genetic dissection using an “immortalized F_2_” population [46].

Opposite to genetic mechanisms of heterosis, reports on the genetic basis of inbreeding depression were inconsistent due to diverse materials in different experiments [47–54]. Wright [51] suggested that recessive or partially recessive deleterious effects of alleles were the most important cause of inbreeding depression. However, overdominance and epistasis were suggested as the primary genetic basis of inbreeding depression in rice [48–49]. Dominance effect was clearly a cause of inbreeding depression for yield while dominance and epistasis effects were the genetic basis of heterosis in soybean [53]. To understand the genetic basis of inbreeding depression in rice, Li and Luo et al. [48–49] reported that hybrid breakdown (part of inbreeding depression) in an intersubspecific F_4_ population and recombinant inbred lines and it’s two BC and two testcross hybrid populations derived from crosses between the RILs and their parents, and these results implied epistasis as a genetic basis of inbreeding depression. Li et al. [54] reported that hybrid breakdown (part of inbreeding depression) in an intersubspecific F_4_ population was largely due to additive epistatic loci, which implies epistasis as a genetic basis of heterosis.

In the present research, the main objective was to explore the genetic basis of heterosis using the populations of F_2: 3_ and F_2: 4_ generations in Upland cotton by dissecting QTLs effects at both single- and two-locus levels. These results are expected to improve our understanding of the genetic basis of heterosis in cotton which will promote cotton hybrid production for lint yield.

## Materials and Methods

### Ethics Statement

No permits were required to conduct the field research or genotyping analyses. The field studies did not involve endangered or protected species.

### Plant Materials

As described previously [17, 20, 55–56], the hybrid of Upland cotton ‘Xinza No. 1’ from the cross of ‘GX1135’ (P_1_) and ‘GX100-2’ (P_2_) was bred by the Guoxin Seed Company (Hebei Province, China), and the hybrid was released as a cultivar in Anhui Province in 2006. In the present study, F_1_ seeds were developed from a manual emasculated cross between P_1_ and P_2_ grown in Hainan Province in winter 2006, and the F_1_ seeds were then grown at the Xinzhou Cotton Breeding Station (Wuhan, 30°34'N, 114°16'E) in April 2007. The genotype of the F_1_ individuals was distinguished by a codominant molecular marker, and one F_1_ individual with the confirmed genotype was self-pollinated to produce F_2_ seeds. A total of 256 randomly selected F_2_ seeds were cultured in nutrient solution [57] in green house at China Agricultural University (Beijing) in October 2007. All the F_2_ seedlings were transported by air and transplanted at the Hainan South Propagation Station (Sanya, Hainan Province) to produce F_3_ seeds by self pollination. The F_3_ family lines were bulk self-pollinated to produce F_4_ seeds. A population of 173 F_2:3_ family lines were planted with their parents and the only one F_1_ individual as, and a population of 173 F_2:4_ family lines were planted with their parents and F_2_ plants as controls in 2009.

### Field Planting and Traits Examination

Field trials of the F_2: 3_ and F_2: 4_ populations were conducted at two locations, Quzhou Experimental Station of China Agricultural University at Handan City, Hebei Province (36°78'N, 114°92'E) and Hejian Guoxin Cotton Breeding Experiment Station at Cangzhou City, Hebei Province (38°43'N, 116°09'E) during 2008 and 2009. The two parents GX1135 (P_1_) and GX100-2 (P_2_) and their F_1_ hybrid, ‘Xinza No. 1’, were also included as controls in the field tests. Field experiments followed a randomized complete block design with two replications at each location. Plants were planted in two row plots with 14 plants each. Plots were 4 m in length with 80 cm row spacing for the experiment at Handan and 4 m in length and 60 cm row spacing for the experiment at Cangzhou. Plants were spaced 30 cm in rows. Field management followed conventional standard field practices. Seedlings of approximate 25 days old were transplanted to fields in 2008. The seeds of F_2: 4_ were planted by sowing directly in 2009. Bolls from seven and five consecutive plants in the middle of each plot were sampled at Handan and Cangzhou, respectively. Boll samples were ginned for seed-cotton yield (SY, t/ha), lint yield (LY, t/ha), bolls/plant (BNP), boll weight (BW, g), lint percentage (LP, %). Traits examined included yield per plant measured as the seed cotton weight of all bolls of the individual. The remaining bolls in each plot were collected for yield estimation. Mid-parent heterosis (MPH) was calculated as MPH = (F_1_-M)/M × 100, M = (P_1_ + P_2_)/2. Where M is the mean yield of both parents and P_1_ and P_2_ are maternal and paternal yield, respectively. Hybrid breakdown (part of inbreeding depression) in an intersubspecific F_4_ population was analyzed by the method provided previously by Li et al. [54].

### DNA Isolation and Genotype Analysis

Young leaves were collected from labeled F_2_, P_1_, P_2_, and F_1_ individuals, frozen in liquid nitrogen, and stored at -80°C. DNA was individually extracted as described by Paterson et al. [58]. A total of 16,405 SSR primer pairs were used to screen for polymorphic markers between parents. Among these primers, the sequences of 13,468 pairs including BNL, NAU, TM, JESPER, CIR, HAU, CM, MUSS, MUSB and MUCS primers were downloaded from Cotton Microsatellite Database (CMD, http://www.cottonmarker.org). The detailed information of these primers was described in literature [6–9, 14, 59]. The remaining 2,937 pairs of primers were designed and developed from DNA sequence library [60]. The polymorphic markers identified were used to genotype individuals of F_2_ population.

### Map Construction and QTL Analysis

MAPMAKER 3.0 [61] was employed to construct a genetic linkage map. Assignment of linkage groups to chromosomes was made based on previously chromosome-anchored SSR markers [6, 11–12, 18, 62]. When no chromosome reference was available, the linkage group was described as “un××” with “××” referring to its serial number. QTLs were detected by composite interval mapping as described by Zeng [63] using software of WinQTL Cartographer 2.5 [64]. A stringent LOD threshold of 3.0 was used to declare suggestive QTL [65], whereas the same QTL in another environment with LOD of at least 2.0 was considered to be a common QTL, as described by Shen et al. [66]. The graphic representation of the linkage group and QTL marked were created by Map Chart 2.2 as described by Voorrips [67].

### Analysis, Confirmation of Digenic Interaction and Components Partition

Analysis of digenic interactions and further confirmation followed the methods described by Yu et al. [43] and Hua et al. [42]. The detected epistasis was confirmed by a randomization test conducted to identify those interactions more likely to be ‘really’ significant. In doing so, the entry order of the trait data in the analysis was randomly permutated and the F-statistic values were recalculated for the digenic interactions using the same marker data. This procedure was repeated 1000 times, and the resulting 1000 F-values were compared with the F-statistic values from the original data. If no more than one F-value from the random permutations was larger than the F-statistic value from the original data, the digenic interaction was regarded to be significant [41]. Significant interactions were further partitioned into three components, each specified by a single degree of freedom: AA (additive × additive), AD/DA (additive × dominance or dominance × additive), and DD (dominance × dominance). The significance for each term was assessed in an orthogonal contrast test using the statistical package STATISTICA [68].

## Results

### Measurements of Yield and Yield Components in F_2: 3_ and F_2: 4_ Populations and Mid-Parent Heterosis of Hybrid ‘Xinza No. 1’

The measurements of yield and yield components including boll number per plant (BNP), boll weight (BW), and lint percentage (LP) were made for hybrids and their parents in F_2: 3_ and F_2: 4_ populations (S1 File). Data are listed in Table 1 and illustrated in S1 Fig. The yield traits varied widely in the populations during the two-year experiments (S1 Fig). Transgressive segregation of both directions was observed for all yield traits in F_2: 3_ and F_2: 4_ populations. Seed cotton yield (SY) and lint yield (LY) of the F_2: 4_ population grown at Handan were much lower than that of the same population grown at Cangzhou, probably due to drought conditions at Handan which caused early aging during experiments. Lint yield was significantly correlated with all other yield traits and seed-cotton yield was significantly correlated with all the traits except lint percent. The correlation coefficients between different traits varied greatly from -0.23 to 0.97. Highest correlations were detected for bolls per plant vs. lint yield, 0.55 and 0.56 at Handan and Cangzhou respectively, in F_2: 3_ populations (Table 2). Significant heterosis for yield was detected in hybrid ‘Xinza No. 1’ with 56% and 62% mid-parent heterosis (MPH) for seed cotton yield and lint yield, respectively. In the F_2_ population, MPH values were 38.1% and 43.3% for seed yield and lint yield, respectively.

**Table 1:** Yield and yield components in F_2: 3_, F_2: 4_ populations and mid-parent heterosis in F_1_ and F_2_ generations.

Trait	Mean	SD	Min	Max	GX1135	GX100-2	Xinza No.1	Mid-parent heterosis (%)
S1/N1^a^								
SY^b^	4.16/2.70	0.80/0.49	2.17/1.47	6.65/4.16	3.66	3.75	5.78	56.23
LY	1.69/1.06	0.32/0.21	0.88/0.58	2.71/1.60	1.55	1.37	2.37	62.01
BNP	15.05/20.20	2.23/3.06	10.17/13.75	20.21/29.67	18.11	16.15	20.34	18.74
BW	5.27/5.95	0.56/0.63	3.34/4.54	6.67/7.56	5.45	5.67	5.57	0.18
LP (%)	40.56/39.16	1.90/1.98	34.47/33.32	45.25/46.05	42.49	36.49	40.91	3.60
S2/N2^a^							F_2_	
SY^b^	1.67/3.68	0.26/0.67	0.98/1.84	2.50/5.62	2.92	2.23	3.55	38.13
LY^b^	0.62/1.53	0.14/0.28	0.35/0.73	0.93/2.32	1.17	0.82	1.42	43.31
BNP	15.24/17.35	1.96/3.13	11.29/10.57	20.93/27.45	21.99	13.58	24.86	39.80
BW	4.29/5.14	0.41/0.47	3.01/3.70	5.20/6.29	4.67	4.60	4.98	7.50
LP (%)	36.91/41.58	1.73/1.88	32.89/34.19	42.00/45.79	40.17	36.66	40.10	4.38

SY: seed cotton yield; LY: lint yield; BNP: bolls per plant; BW: boll weight; LP: lint percent

^a^ S1/N1: F_2:3_ family (S1: 2008Handan, N1:2008Cangzhou), S2/N2: F_2:4_ family(S2: 2009Handan, N2: 2009Cangzhou) (the same below)

^b^ t/ha

Results in each cell are presented as S/N

**Table 2:** Correlation between yield and yield components in F_2: 3_ and F_2: 4_ populations.

Trait	Population	Seed cotton yield	Lint yield	Bolls/plant	Boll weight
Lint yield	S1/N1^a^	0.97**/0.97**			
	S2/N2^a^	0.96**/0.97**			
Bolls/plant	S1/N1	0.50**/0.55**	0.55**/0.56**		
	S2/N2	0.18*/0.37**	0.23**/0.40**		
Boll weight	S1/N1	0.25**/0.30**	0.24**/0.23**	0.01/0.04	
	S2/N2	0.62**/0.27**	0.61**/0.21**	0.00/-0.03	
Lint percent (%)	S1/N1	-0.09/0.12	0.16*/0.36**	0.16*/0.17*	-0.00/-0.23**
	S2/N2	0.12/-0.05	0.40**/0.19**	0.25**/0.14	0.13/-0.23**

*, ** Significant at probability of 0.05 and 0.01 respectively.

### Linkage Map and QTL Mapping for Yield and Yield Components

A total of 450 polymorphic markers, screened from 16,405 pairs of SSR primer between parents, were used to construct a linkage group (S2 File). The linkage map was obtained with 421 loci linked into 49 groups leaving 29 loci unlinked. The map spanned 3814 cM with an average distance of 8.9 cM between adjacent markers, accounting for 73% of the entire tetraploid genome. Forty-eight of the 49 linkage groups were assigned to 26 chromosomes. The remaining one linkage group could not be associated with any chromosome, and the one group was tentatively named as ‘Un 1’.

A total of 67 QTLs controlling yield and yield components were identified in F_2: 3_ and F_2: 4_ generations using composite interval mapping. The data are given in Table 3 and S2 Fig. There were 38 QTLs identified in F_2: 3_ generation, among which seven were major QTLs detected in both environments. There were 49 QTLs identified in F_2: 4_ generation, among which, six were major QTLs detected in both environments.

**Table 3:** QTLs for yield and yield-components in F_2: 3_ and F_2: 4_ populations identified using composite interval mapping.

Trait	QTLs	Env	Chr	Marker flanking		Position (cM)	LOD	A^a^	D^b^	D/A	Var%^c^
SY(S1N1^e^)	***qSY-chr11-1*** *	S1	11	BNL2895	TMB628	0.62	2.78	-6.39	2.18	0.34	6.08
	***qSY-chr12-1*** *	S1	12	CGR5193	DPL252	14.73	3.59	-2.16	-5.28	-2.44	7.62
	***qSY-chr14-1*** *	S1	14	DPL565	CGR6683	78.05	5.18	5.78	3.32	0.58	12.71
		N1	14	DPL565	CGR6683	80.05	3.15	6.83	-0.73	-0.11	7.00
	***qSY-chr26-1***	S1	26	CGR6880	CGR5452	63.34	2.35	-4.35	-1.90	-0.44	5.24
		N1	26	CGR6880	CGR5452	63.34	3.39	-8.74	0.67	0.08	7.59
	*qSY-chr5-1*	N1	5	TMB1489	DC40111	6.26	3.33	-8.16	2.91	0.36	6.68
	*qSY-chr13-1*	N1	13	NAU2893	GH157	125.04	5.44	10.60	-5.08	-0.48	11.54
	*qSY-chr23-1*	S1	23	GH499	SHIN0830	0.01	3.23	-6.57	5.26	0.80	6.54
SY(S2N2^e^)	***qSY-chr11-1*** *	N2	11	TMB628	GH316	1.73	2.02	-5.07	4.64	0.92	4.50
	***qSY-chr12-1*** *	S2	12	CGR5193	DPL252	18.73	2.75	4.08	-2.29	-0.56	5.26
	***qSY-chr14-1*** *	S2	14	CGR6683	HAU1057	83.06	2.22	1.25	0.10	0.08	3.64
	*qSY-chr5-2*	S2	5	HAU911	HAU746	39.08	7.25	-3.08	0.22	0.07	13.57
	*qSY-chr8-1*	S2	8	NAU4045	CGR6129	38.52	3.66	2.46	-0.42	-0.17	7.50
	*qSY-chr10-1*	S2	10	NAU3404	GH199	64.97	3.14	-1.11	-1.03	-0.93	5.50
	*qSY-chr11-2*	S2	11	CGR5602	DPL050a	73.77	10.15	1.95	1.95	1.00	23.13
	*qSY-chr12-2*	S2	12	CGR5111	BNL3261	2.96	4.65	-0.75	-1.77	-2.37	7.97
LY (S1N1)	***qLY-chr5-1*** *	N1	5	TMB1489	DC40111	6.26	3.13	-3.32	1.38	0.41	5.98
	***qLY-chr5-2*** *	S1	5	HAU1315	NAU4034	40.67	2.52	-1.04	-1.36	-1.30	4.82
	***qLY-chr8-1*** *	N1	8	NAU1356	DPL790	18.01	2.58	3.37	-0.86	-0.26	6.28
	***qLY-chr26-1***	S1	26	CGR6880	CGR5452	63.34	3.18	-1.58	-1.55	-0.98	6.80
		N1	26	CGR6880	CGR5452	63.34	5.79	-4.72	0.36	0.08	12.64
	*qLY-chr13-1*	N1	13	GH157	BNL1495	126.06	5.10	3.85	-0.86	-0.22	10.01
LY (S2N2)	***qLY-chr5-1*** *	N2	5	DPL022	TMB0865	0.01	2.46	2.76	-2.34	-0.85	5.22
	***qLY-chr5-2*** *	S2	5	HAU911	HAU746	39.08	4.74	-0.98	-0.01	-0.01	9.23
		N2	5	HAU911	HAU746	39.08	2.19	-1.18	-1.13	-0.96	4.95
	***qLY-chr8-1*** *	S2	8	DPL790	NAU4045	28.09	2.04	0.56	0.22	0.38	4.70
	*qLY-chr11-1*	S2	11	CGR5602	DPL050a	77.77	7.49	0.60	0.76	1.28	16.21
	*qLY-chr11-2*	N2	11	CGR5217	DPL131	109.77	3.69	1.76	0.62	0.35	8.53
	*qLY-chr12-1*	S2	12	CGR5111	BNL3261	4.96	3.69	-0.29	-0.66	-2.32	7.16
	*qLY-chr14-1*	S2	14	GH529	CGR5675	20.18	3.06	0.74	0.30	0.41	6.84
	*qLY-chr19-1*	S2	19	DC40122	NAU1221a	45.80	3.68	-1.03	0.60	0.58	6.85
BNP (S1N1)	***qBNP-chr14-1*** *	S1	14	DPL502	NAU2960	13.58	2.29	0.83	-0.98	-1.18	4.78
	***qBNP-chr19-1*** *	S1	19	NAU3437	GH616	12.01	2.04	-0.96	1.10	1.15	8.21
	***qBNP-chr21-1*** *	N1	21	DPL050b	NAU3695	17.09	2.58	-1.21	0.50	0.41	6.27
	***qBNP-chr5-1***	S1	5	NAU2140	NAU792	88.07	2.06	-0.50	1.02	2.04	5.24
		N1	5	NAU6240	DPL591	63.82	3.25	1.31	-2.13	-1.63	12.15
	*qBNP-chr8-1*	N1	8	NAU4045	CGR6129	34.52	3.06	1.67	-0.96	-0.57	6.81
	*qBNP-chr14-2*	S1	14	BNL2485	CIR228	2.01	3.04	0.66	-1.22	-1.85	7.50
	*qBNP-chr24-1*	N1	24	DPL551	GH273	23.47	3.27	-1.06	-0.21	-0.20	8.37
	*qBNP-chr24-2*	S1	24	CGR5423	SHIN1076	56.71	4.39	-0.64	-0.55	-0.86	9.78
BNP (S2N2)	***qBNP-chr14-1*** *	S2	14	DPL502	NAU2960	17.58	2.72	0.91	-0.72	-0.79	6.87
	***qBNP-chr19-1*** *	N2	19	NAU3437	GH616	4.01	2.43	-0.85	0.97	1.14	20.23
	***qBNP-chr21-1*** *	S2	21	NAU965	CGR6525	35.99	4.66	-1.01	0.79	0.79	10.13
		N2	21	NAU965	CGR6525	37.99	3.31	-1.30	1.74	1.34	8.65
	*qBNP-chr1-1*	N2	1	CIR307	HAU1417b	116.44	3.98	0.95	1.16	1.22	13.46
	*qBNP-chr11-1*	N2	11	CGR5808	CGR5217	101.47	3.03	1.46	-1.16	-0.79	8.79
	*qBNP-chr12-1*	S2	12	DPL252	DC20127	33.65	5.13	-0.56	-0.80	-1.43	16.63
	*qBNP-chr20-1*	N2	20	GH451	CGR5548	23.57	3.39	-0.08	-1.47	-17.31	8.86
	*qBNP-chr22-1*	S2	22	CGR5806	CGR6410	25.49	3.26	-0.21	1.07	5.11	6.96
	*qBNP-chr23-1*	S2	23	GH327	NAU3100	6.01	2.00	0.42	0.35	0.83	4.49
BW (S1N1)	***qBW-chr5-1*** *	S1	5	BNL3447	GH260	26.01	2.40	-0.24	0.17	0.71	7.54
		N1	5	BNL3447	GH260	26.01	2.50	-0.18	-0.01	-0.06	8.14
	***qBW-chr5-2*** *	N1	5	NAU2865	GH388	37.34	4.61	-0.25	-0.09	-0.35	9.69
		S1	5	HAU1603	TMB1296	38.92	2.70	-0.23	0.07	0.30	5.94
	***qBW-chr6-1*** *	S1	6	DPL124	HAU1460	52.74	2.90	0.20	-0.04	-0.21	7.05
	***qBW-chr13-1*** *	N1	13	BNL1495	DPL687	130.09	2.91	0.08	-0.32	-4.06	6.18
	***qBW-chr25-1*** *	N1	25	HAU1382	BNL3594	8.01	2.38	0.13	-0.32	-2.57	6.59
	*qBW-chr2-1*	S1	2	MGHE24	COT064	65.20	3.14	-0.20	-0.03	-0.12	6.97
	*qBW-chr7-1*	S1	7	GH308	GH474	22.08	3.10	0.23	-0.25	-1.12	6.21
	*qBW-chr15-1*	S1	15	DPL0752	CGR6847	103.01	3.46	0.08	0.20	2.40	7.72
BW (S2N2)	***qBW-chr5-1*** *	N2	5	BNL3447	GH260	32.01	2.63	-0.14	-0.04	-0.29	7.20
	***qBW-chr5-2*** *	S2	5	GH388	HAU1603	38.59	2.05	-0.11	-0.01	-0.10	3.84
	***qBW-chr6-1*** *	S2	6	CGR6749	DPL124	50.32	2.69	0.06	0.07	1.23	5.13
	***qBW-chr13-1*** *	S2	13	DPL687	DPL286	134.49	3.65	0.23	-0.24	-1.03	7.75
	***qBW-chr25-1*** *	N2	25	BNL3594	CGR6864	15.09	2.90	0.20	-0.20	-0.99	6.28
	***qBW-chr4-1***	S2	4	BNL3990	BNL0530	38.01	2.86	-0.17	0.09	0.54	5.30
		N2	4	BNL3990	BNL0530	38.01	3.27	-0.23	0.14	0.63	7.23
	***qBW-chr6-2***	S2	6	DPL590	BNL3567	71.65	2.64	0.08	0.06	0.82	5.47
		N2	6	DPL590	BNL3567	63.65	2.04	0.04	0.12	2.77	5.11
	*qBW-chr6-3*	S2	6	GH119	BNL3650	87.26	3.20	0.14	0.00	-0.03	6.44
	***qBW-chr11-1***	S2	11	BNL3171	CGR5808	87.95	6.70	0.23	-0.04	-0.18	14.11
		N2	11	DPL050a	HAU423	85.10	2.68	0.21	-0.18	-0.85	6.68
	*qBW-chr11-2*	N2	11	CGR5808	CGR5217	99.47	3.74	0.23	-0.14	-0.61	10.44
	*qBW-chr12-1*	S2	12	CGR5111	BNL3261	2.96	3.93	-0.14	-0.03	-0.19	7.96
	*qBW-chr14-1*	S2	14	NAU3839	BNL3033	83.89	5.16	0.10	0.12	1.13	10.16
	*qBW-chr24-1*	S2	24	SHIN0272	DPL551	0.01	3.00	0.00	0.15	39.48	22.13
LP(S1N1)	***qLP-chr1-1*** *	S1	1	CIR307	HAU1417b	94.44	3.75	0.88	-0.67	-0.76	8.12
	***qLP-chr4-1*** *	S1	4	BNL0530	JESP295	46.02	2.83	0.78	-0.67	-0.86	5.37
	***qLP-chr5-1*** *	S1	5	GH260	NAU6240	46.92	3.76	0.95	-0.77	-0.81	9.33
		N1	5	GH260	NAU6240	42.92	6.10	1.34	-0.75	-0.56	16.7
	***qLP-chr6-1*** *	S1	6	NAU3490	DC40076	24.01	3.41	-0.03	0.83	31.09	14.32
	***qLP-chr7-1*** *	S1	7	NAU1043	SHIN0376	82.41	6.69	1.06	0.07	0.07	14.5
	***qLP-chr13-1*** *	N1	13	DPL687	DPL286	140.49	3.27	0.56	0.47	0.84	7.65
	***qLP-chr26-1*** *	N1	26	NAU5072	BNL2495	88.09	2.95	-0.49	-0.40	-0.82	6.43
	*qLP-chr2-2*	N1	2	TMB1268	JESP304	31.61	3.05	-0.86	0.59	0.68	5.95
	*qLP-chr11-1*	N1	11	CGR5602	DPL050a	73.77	3.20	0.86	-0.16	-0.19	8.36
	*qLP-chr20-1*	N1	20	HAU1314	HAU748	0.01	3.57	-0.83	0.97	1.16	7.02
LP (S2N2)	***qLP-chr1-1*** *	S2	1	DC40175	CIR307	85.83	3.00	0.67	-0.24	-0.36	7.84
	***qLP-chr4-1*** *	S2	4	BNL0530	JESP295	50.02	2.38	0.64	-0.79	-1.24	6.61
		N2	4	BNL0530	JESP295	48.02	2.73	0.57	-0.92	-1.61	6.24
	***qLP-chr5-1*** *	N2	5	GH260	NAU6240	38.92	6.68	1.20	-0.56	-0.47	16.18
	***qLP-chr6-1*** *	S2	6	NAU3490	DC40076	18.01	2.14	-0.88	1.07	1.21	8.16
	***qLP-chr7-1*** *	S2	7	NAU1043	SHIN0376	82.41	4.05	0.81	-0.07	-0.09	9.17
	***qLP-chr13-1*** *	N2	13	DPL687	DPL286	140.49	4.33	0.54	0.68	1.26	10.7
	***qLP-chr26-1*** *	N2	26	CER144	NAU5072	79.42	2.47	-0.89	0.48	0.54	7.36
	*qLP-chr2-1*	N2	2	CGR6695	DPL217	39.71	3.14	-0.91	0.82	0.90	7.54
	*qLP-chr6-1*	S2	6	HAU1460	DPL127	60.53	3.22	-1.21	0.34	0.28	6.60
	*qLP-chr12-1*	N2	12	NAU3519	NAU3860	34.01	3.64	0.25	1.46	5.92	13.88
	*qLP-chr13-1*	S2	13	COT009	DPL308	96.39	2.09	0.71	-0.20	-0.28	4.80
	*qLP-chr19-1*	S2	19	NAU1042	NAU797	44.42	3.17	-0.78	0.64	0.82	6.81

* Common QTLs identified in the two generations

^a^ Additive effects; positive values of the additive effects indicate increase of traits from alleles of GX1135; negative values of the additive effects indicate decrease of traits from alleles of GX100-2

^b^ Dominance effects; positive values of the dominance effect indicate that heterozygotes have higher phenotypic values than the respective means of two homozygotes, and negative values indicate that heterozygotes have lower values than the means of the two homozygotes

^c^ Var%, phenotypic variation explained by a single QTL

For seed cotton yield, a total of 12 QTLs were identified in two generations, of those three QTLs were detected simultaneously in the two generations. Seven QTLs were identified in F_2: 3_ generation, accounting for 5.2% to 12.7% of total variance, and eight QTLs were identified in F_2: 4_ generations, accounting for 3.6% to 23.1% of total variance, respectively. Among them, two QTLs, *qSY-chr26-1* and *qSY-chr14-1*, were identified at both Handan and Cangzhou locations in F_2: 3_ generation. Three QTLs, *qSY-chr11-1*, *qSY-chr12-1* and *qSY-chr14-1*, were detected in both F_2: 3_ and F_2: 4_ generations. Six QTLs identified in F_2: 3_ generation showed negative additive effects (Table 3) and the alleles from female parent GX1135 increased phenotypic variation (S2 Fig). Three QTLs in F_2: 3_ generation showed positive additive effects and the alleles from GX100-2 increased phenotypic variation. In F_2: 3_ and F_2: 4_ generations, eight QTLs showed negative dominance effects and the remaining nine QTLs showed positive dominance effects.

For lint yield, a total of 10 QTLs were identified in two generations, among which three QTLs were detected simultaneously in the two generations. Five and eight QTLs were identified in F_2: 3_ and F_2: 4_ generations, respectively, accounting for 4.8% to 12.6% and 4.7% to 16.2% of total phenotypic variance in the two generations, respectively. Four QTLs among these were identified simultaneously in more than two environments. Two QTLs, *qLY-chr26-1* for lint yield and *qSY-chr26-1* for seed cotton yield, were mapped to the same interval on chromosome 26 in the same environments (S2 Fig).

For bolls per plant, 14 QTLs were found in two generations, among which, three QTLs were detected simultaneously in the two generations. Eight and nine QTLs were identified in F_2: 3_ and F_2: 4_ generations, respectively, accounting for 4.8% to 12.2% and 4.5% to 20.2% of total phenotypic variance in the two respective generations. Four of them were detected simultaneously in more than two environments. Five and six QTLs with negative additive effects were detected in F_2: 3_ and F_2: 4_ generations, respectively. The alleles from female parent GX1135 increased phenotypic variation of lint yield. The remaining eight QTLs showed positive additive effects in the two generations. For the effects of these QTLs, the alleles from GX100-2 increased phenotypic variation. There were six and four QTLs identified with negative dominance effects in F_2: 3_ and F_2: 4_ generations, respectively. The results suggested that heterozygotes would have lower values than the two homozygotes for these QTLs. The remaining nine QTLs showed positive dominance effects indicating that heterozygotes would have higher phenotypic values than the two respective homozygotes for the effects of these nine QTLs.

A total of 15 QTLs referring boll weight were resolved in two generations, among which five QTLs were detected simultaneously in the two generations. Eight and 12 QTLs were identified in F_2: 3_ and F_2: 4_ generations, respectively, explaining 5.9% to 9.7% and 3.8% to 22.1% of phenotypic variance in the two respective generations. There were two QTLs (*qBW-chr5-1* and *qBW-chr5-2*) simultaneously detected in F_2: 3_ and F_2: 4_ generations with negative additive effects. The alleles from the female parent GX1135 increased phenotypic variation for the effects of these QTLs. There were seven and eight QTLs showing negative dominance effects in F_2: 3_ and F_2: 4_ generations, respectively. There were three and eight QTLs in F_2: 3_ and F_2: 4_ generations, respectively, showing positive dominance effects.

For lint percent, a total of 15 QTLs were identified in two generations, among which seven QTLs were observed simultaneously in the two generations. Ten and 12 QTLs were identified in F_2: 3_ and F_2: 4_ generations, respectively, explaining 5.4% to 14.3% and 4.8% to 16.2% of phenotypic variance in the two respective generations. Among them, there were seven QTLs detected in more than two environments. There were four and five QTLs in F_2: 3_ and F_2: 4_ generations, respectively, with negative additive effects. For these QTLs, alleles from the female parent GX1135 increased phenotypic variation (Table 3). The remaining 15 QTLs in the two generations showed positive additive effects with the alleles from female parent GX100-2 increasing phenotypic variation. There were six and six QTLs detected in F_2: 3_ and F_2: 4_ generations, respectively, with negative dominance effects. The remaining 12 QTLs in the two generations showed positive dominance effects.

### Dominance and Overdominance Effects of QTL

A locus is regarded as having overdominance if the ratio of the estimated dominance to the absolute value of additive effect is larger than one. Similarly, a locus is regarded as having partial dominance if the ratio is between 0 and 1 [34]. A total of 32 and 36 QTLs in F_2: 3_ and F_2: 4_ populations, respectively, showed partial dominance with their ratios ranging from 0 to 1. One QTL was detected for seed cotton yield showing dominance in F_2: 4_ generation. Thirteen and 19 QTLs in F_2: 3_ and F_2: 4_ populations, respectively, showed overdominance with ratio greater than 1. Partial dominance occurred more frequently than overdominance and dominance. More than half of the QTLs listed in Table 3 showed varying degrees of negative dominance indicating that heterozygosity does not necessarily favor over homozygosity for yield. Similar results were reported previously in a study in an elite rice hybrid [41].

### Relationship between Marker Heterozygosity and Performance

Correlation coefficients were not significant between heterozygosity of the marker genotypes and the performance of these genotypes in terms of yield and yield components in four environments (Table 4). These results implied difficulty in prediction of agronomic performance based on heterozysity of marker genotypes. One possibility for the low correlation is that only a portion of the 362 marker loci is related to the performance of those traits. These results were consistent with previous reports [41, 69].

**Table 4:** Correlation coefficients between genotypic heterozygosity and trait performance in F_2: 3_ and F_2: 4_ populations.

Trait	S1	N1	S2	N2
Seed cotton yield	0.03	-0.07	0.00	-0.01
Lint yield	0.02	-0.07	-0.01	0.01
Bolls/plant	0.05	-0.03	0.10	0.00
Boll weight	-0.05	-0.10	-0.06	-0.03
Lint percent	-0.04	0.01	-0.05	0.06

### Identification and Verification of Digenic Interactions

The number of digenic interactions identified by two-way ANOVA for lint yield and yield components are given in Table 5. There were a total of 65,341 tests using 362 co-dominant markers at the whole genome level. For individual tests at the 0.001 probability level, the expected number of spurious interactions would be 65. The interaction number of the traits analyzed in the four environments ranged from 70 to 234, although the number analyzed for boll weight of F_2: 3_ population at Handan (S1) and the number analyzed for lint percent of F_2: 4_ population at Cangzhou (N1) was 64 and 65, respectively. To further assess the likelihood of the interactions identified above as chance events, each of the declared significant interactions was subjected to randomization tests [41]. The numbers of significant interactions decreased after randomization tests, and the reductions were more than the expected numbers based on chance events in all the cases. Current result indicated that the randomization test was highly stringent in identification of the significant interactions. For example, the number of significant interactions identified for lint yield in F_2: 3_ populations was 109 and 106 at the two respective experimental sites, and reduced to 97 and 93, respectively, after randomization test. In F_2: 4_ populations, the number of significant interactions was from 99 and 160 at the two respective experimental sites, and reduced to 93 and 149, respectively, after randomization tests (Table 5). The interactions that survived randomization tests may therefore be regarded as the minimum number of significant interactions for each trait at the 0.001 probability level. In this way, large numbers of significant digenic interactions in the two populations were confirmed.

**Table 5:** Number of significant interactions for yield and yield components detected at 0.001 probability by permutation tests of all possible two loci combinations in hybrids evaluated at Handan (S1) and Cangzhou (N1.).

Trait	Whole-genome searching		Common in F_2: 3_	Confirmed by randomization test			Whole-genome searching		Common in F_2: 4_	Confirmed by randomization test		
	S1	N1		S1	N1	Common	S2	N2		S2	N2	Common
Lint yield	109	106	1	97	93	1	99	160	0	93	149	0
Bolls/plant	82	234	1	77	211	1	107	82	0	104	76	0
Boll weight	82	64	0	77	60	0	116	115	2	104	110	2
Lint percent	108	70	6	98	61	6	116	78	1	107	65	1

In the F_2: 3_ population, 97 digenic interactions for lint yield were detected at location of S1, and 93 digenic interactions were detected at location of N1 with one digenic interaction detected at both of the two locations. For the yield component of bolls per plant, 77 and 211 digenic interactions were detected at Handan (S1) and Cangzhou (N1), respectively, with one digenic interaction detected at both locations. For boll weight, 77 digenic interactions were detected at location of Handan, and 60 digenic interactions were detected at location of Cangzhou. There was no common digenic interaction detected at both locations for this yield component. For the yield component of lint percent, 98 digenic interactions were detected at location of Handan, and 61 digenic interactions were detected at location of Cangzhou with six common digenic interactions detected at both locations.

Interactions were partitioned by orthogonal contrasts into significant genetic components, and the significant genetic components were further confirmed by randomization tests (Table 6). In general, among two-locus interaction components, AA interactions for lint yield and yield components had the highest frequencies.

**Table 6:** Summary of the significant (p≤0.001) interactions detected for lint yield and yield components by permutation tests of all possible two loci combinations.

Trait	Interaction	S1^a^	N1^a^	Common in F_2: 3_
Lint yield	Positive pairs	97	93	1
	AA	69	62	0
	AD/DA	48	34	0
	DD	6	19	0
Bolls/plant	Positive pairs	77	211	1
	AA	56	149	0
	AD/DA	29	100	0
	DD	8	12	0
Boll weight	Positive pairs	77	60	0
	AA	53	34	0
	AD/DA	30	36	0
	DD	8	4	0
Lint percent	Positive pairs	98	61	6
	AA	59	29	2
	AD/DA	51	38	2
	DD	12	10	0

These results indicated that all genetic components, AA, AD/DA, DD, existed in F_2: 3_ generation for all traits detected with significant interactions. In contrast, DD interactions occurred with the lowest frequencies and AD/DA occurred with intermediate frequencies.

### Effects of Epistatic Interaction

Significant heterosis was detected for boll number per plant, and this yield component contributed directly to lint yield [3]. This yield component was also identified as the most important yield component to lint yield in recent studies [70–71]. This yield component was used as an example in this study to illustrate digenic effects in F_2: 3_ population.

Effects of digenic interactions for the best double homozygotes, i.e., homozygotes of two-locus combinations with significant AA, are given in Table 7. A total of 56 two-locus combinations with significant AA, 33 interactions of complementary two-locus homozygotes (11/22 or 22/11) had distinct advantages over the means of two parental genotypes (11/11 or 22/22) (Table 7). Among them, twenty-two cases were for complementary two-locus homozygotes, 11/22, and eleven cases were for complementary two-locus homozygotes, 22/11. Among the 56 epistasis, sixteen and six cases were the best loci combination from homozygotes of 22/22 and 11/11, respectively. Occasionally, single heterozygotes (six and one cases out of 27 tests for 11/12 and 22/12, respectively) could be comparable to the best two-locus genotypes. Generally, the heterotic values of the two-locus combinations (11/22 or 22/11, eight cases out of 27 tests) and homozygotes (22/22, 11 cases out of 27 tests) with significant AD/DA interactions had larger heterosis effects than single heterozygotes (11/12, 22/12) (Table 8). These results indicated the complementary effects in two-locus homozygotes. In order to test the hypothesis that double heterozygotes (12/12) have advantage over single heterozygotes, two-locus combinations with significant DD effects were compared with two locus heterozygotes (Table 9). Most of the best two-locus genotypes were homozygous at one locus and heterozygous at the other locus (11/12 and 22/12). Advantage of the double heterozygotes over single heterozygotes was not observed. In summary of these results, the complementary two-locus homozygotes (11/22 or 22/11) frequently showed large effects in heterosis. Single heterozygotes (11/12 and 22/12) and homozygotes (22/22) were the best two-locus genotypes for yield in a few cases while in no case did double heterozygotes (12/12) showed larger effects of heterosis than double homozygotes and single heterozygotes.

**Table 7:** The best and the worst double homozygotes in each of the two loci combinations showing significant AA interactions for bolls/plant in F_2: 3_ population.

Locus 1^a^	Locus 2^a^	Var%	Genotype^b^	Over midparent^c^	Over GX1135^d^	Over 12/12^e^	Best genotype^f^	Worst genotype^g^
BNL3567(6)	NAU2715(26)	8.21	22/22	1.20	2.40*	2.61*	22/22	11/22
BNL569(13)	CGR5217(11)	6.11	11/22	3.00*	2.88*	2.41*	11/22	11/12
C2_115(12)	CGR6695(2)	5.48	22/22	1.15	2.29*	1.98*	22/22	22/11
CER098(11)	SHIN0219(13)	5.54	11/22	1.96*	3.16**	1.18	11/12	11/11
CER165(13)	CGR6683(14)	7.5	11/22	3.23	3.01	2.53	11/22	22/22
CER165(13)	HAU1057(14)	5.06	11/22	2.77	2.95*	2.30	11/22	11/11
CER165(13)	NAU3308(14)	6.91	11/22	3.13*	2.91*	2.37	11/22	22/22
CER165(13)	NAU3839(14)	6.98	11/22	3.23	3.01	2.69	11/22	22/22
CGR115(12)	CGR6695(2)	4.99	22/22	1.15	2.29*	1.98*	22/22	22/11
CGR5056(15)	DPL790(8)	6.15	11/11	1.10	0.00	1.63	11/11	11/22
BNL1040(13)	BNL243(18)	4.65	11/22	3.31**	3.27**	3.23**	11/22	11/12
CGR5108(6)	CGR6867(15)	9.8	22/11	3.07**	2.76**	1.98*	22/11	22/22
CGR5108(6)	NAU2894(19)	10.02	11/11	0.15	0.00	1.26**	11/11	11/22
CGR5111(12)	CGR6695(2)	5.17	22/22	0.99	1.97*	1.70	22/22	12/22
CGR5871a(14)	CGR6539(13)	7.07	22/11	3.51*	3.56*	2.34	22/11	11/11
CGR5873(10)	NAU792(5)	4.32	22/22	1.16	2.33*	1.68	22/22	11/22
CGR5951(5)	DPL022(5)	9.63	22/22	1.27	2.53**	2.37**	22/22	22/11
BNL1053(11)	CGR5758(9)	7.09	22/22	0.30	0.59	0.65	22/22	22/11
CGR6017(3)	NAU5428(11)	11.92	22/11	3.14**	3.41**	0.85	22/11	11/11
CGR6129(8)	NAU5428(11)	7.96	11/22	4.12**	3.97*	2.96*	11/22	22/22
CGR6378(15)	NAU4034(5)	7.02	11/22	2.71*	2.70*	1.97	11/22	12/11
CGR6732(13)	HAU1455(14)	6.36	11/22	3.03*	2.70*	2.62*	11/22	22/22
CGR6732(13)	NAU3308(14)	6.03	11/22	3.21*	3.37*	2.71*	11/22	11/11
CGR6864(25)	COT107(12)	8.78	11/22	3.13**	2.80**	0.67	11/22	22/22
CGR6864(25)	GH631(12)	7.38	11/22	3.03**	2.60*	0.64	11/12	22/22
CGR6864(25)	NAU943(12)	8.83	11/22	3.04**	2.84**	0.58	11/22	22/22
CGR6867(15)	HAU1460(6)	6.2	11/22	2.60*	2.43	1.57	11/22	12/11
BNL243(18)	CGR5554(13)	6.38	22/11	3.18**	3.14**	3.01**	22/11	12/22
BNL243(18)	NAU3177(8)	8.63	22/22	0.06	0.12	1.64	22/22	11/22
COT107(12)	DPL282(25)	7.64	11/22	3.29**	2.77**	0.55	11/22	22/22
COT107(12)	HAU250(18)	6.56	11/22	3.29**	3.86**	2.14*	11/22	11/11
DC40052(15)	DPL790(8)	6.96	11/11	1.30	0.00	2.11*	11/11	11/22
DC40129a(9)	NAU2140(5)	9.85	11/22	3.47**	3.59**	1.13	11/22	11/11
DC40183(24)	MUSS059(2)	9.5	22/22	0.56	1.12	1.66*	22/22	11/22
DC40183(24)	NAU2265(2)	7.79	22/22	0.80	1.59	2.03*	22/22	22/11
DC40183(24)	NAU895(2)	7.99	22/22	0.73	1.45	1.90*	22/22	11/22
DPL056a(5)	DPL0847(6)	6.74	11/22	2.96**	2.88*	1.67	11/22	22/22
DPL056a(5)	DPL564(6)	4.38	11/22	3.61**	3.09*	2.71*	11/22	22/22
DPL056a(5)	DPL590(6)	6.23	11/22	3.13**	3.11**	2.04*	11/22	22/22
DPL090(8)	DPL502(14)	7.72	11/22	2.77**	2.86**	2.15*	11/22	22/12
BNL2469(14)	CER165(13)	6.89	22/11	3.23	3.01	2.47	22/11	22/22
DPL282(25)	DPL303(12)	6.51	22/11	3.09**	3.49**	1.14	22/11	11/11
DPL282(25)	GH631(12)	7.36	22/11	3.01**	2.59*	0.13	22/11	22/22
DPL282(25)	NAU943(12)	8.46	22/11	3.16**	2.94**	0.36	22/11	22/22
DPL588(24)	NAU3217(19)	15.82	22/22	0.52	1.04	2.59*	22/22	11/22
DPL591(5)	BNL3661(14)	5.7	22/22	1.22	2.44**	1.75	22/22	11/22
DPL591(5)	MGHE46(9)	6.57	22/11	2.68**	2.81**	0.99	22/11	11/11
BNL2496(17)	BNL3442(11)	5.96	22/22	1.13*	2.26**	1.79**	22/22	11/22
GH220(25)	MGHE34(9)	7.25	22/11	2.63**	2.86**	1.01	22/11	11/11
HAU1314(un5)	NAU792(5)	6.91	11/11	0.30	0.00	0.41	11/11	11/22
HAU190(un1)	SHIN1076(24)	8.51	22/22	1.44**	2.88**	2.30**	22/22	11/22
MGHE58(7)	NAU3217(19)	8.38	11/11	0.78	0.00	3.37**	11/11	11/22
BNL3033(14)	CER165(13)	6.82	22/11	3.23	3.01	2.47	22/11	22/22
NAU1014(11)	NAU748(18)	6.42	11/22	2.21**	3.04**	0.59	11/22	11/11
BNL3085(15)	CGR5108(6)	7.08	11/22	2.26*	1.92*	1.49	11/22	22/22
NAU3519(12)	JESP056(2)	7.03	22/22	0.65	1.30	1.68*	22/22	22/11

^a^ Interaction markers and its chromosomal locations (in parentheses).

^b^ Genotype of the first locus/genotype of the second locus; 11, homozygous for the P1 allele; 22, homozygous for the P2 allele

^c^ Advantage of the best homozygote over the mean of the two parental genotypes.

^d^ Advantage of the best homozygote over GX1135 genotype

^e^ Advantage of the best homozygote over heterozygote genotype.

^f^ The best genotype identified in the nine interaction types.

^g^ The worst genotype identified in the nine interaction types.

* and * * Significant different from zero at p = 0.05 and p = 0.01 levels respectively.

**Table 8:** The best and the worst single heterozygotes in each of the two loci combinations showing significant AD/DA interactions for bolls/plant in F_2: 3_ population.

Locus 1^a^	Locus 2^a^	Var%	Genotype^b^	Over midparent^c^	Over GX1135^d^	Over 12/12^e^	Best genotype^f^	Worst genotype^g^
BNL3447(5)	GH137(16)	5.74	11/12	-0.21	0.18	0.52	22/11	12/11
CER098(11)	SHIN0219(13)	6.44	11/12	2.19**	3.39	1.41*	11/12	11/11
BNL1040(13)	BNL243(18)	5.17	22/12	0.95*	0.92	0.88*	11/22	11/12
CGR5352(18)	DC40052(15)	7.18	11/12	1.18	2.08**	0.17	22/11	11/11
CGR5534(11)	NAU2277(2)	7.06	11/12	0.85	1.11*	1.10*	11/12	11/22
CGR5873(10)	NAU792(5)	5	11/12	0.35	1.51**	0.86	22/22	11/22
CGR5951(5)	TMB1268(2)	10.51	22/12	1.39**	-0.37	0.29	11/22	22/22
CGR6539(13)	CGR6864(25)	10.67	11/12	1.58**	2.57**	0.92	22/11	11/11
DC40183(24)	MUSS059(2)	4.97	11/12	0.10	0.66	1.20*	22/22	11/22
DC40183(24)	NAU895(2)	7.15	11/12	0.07	0.80	1.25**	22/22	11/22
DPL056a(5)	DPL564(6)	7.03	22/12	1.84**	1.31*	0.94	11/22	22/22
DPL124(6)	GH256(11)	8.3	11/12	0.09	1.42**	0.44	22/22	22/12
DPL551(24)	DPL687(13)	8.18	22/12	2.47**	1.53**	2.79**	22/12	22/11
BNL2496(17)	BNL3442(11)	4.77	11/12	-1.16*	-0.03	-0.50	22/22	11/22
GH144(10)	GH388(5)	4.94	11/12	1.25**	2.27**	2.49**	11/12	12/12
C2_115(12)	CGR6695(2)	6.71	12/11	-0.83	0.32	0.00	22/22	22/11
CGR115(12)	CGR6695(2)	6.71	12/11	-0.83	0.32	0.00	22/22	22/11
CGR5111(12)	CGR6695(2)	6.44	12/11	-0.88	0.11	-0.16	22/22	12/22
CGR5554(13)	DPL244(6)	9.11	12/11	0.49	0.83	0.45	11/22	12/22
CGR5554(13)	HAU1460(6)	13.42	12/11	0.55	1.33*	0.54	11/22	12/22
CGR6864(25)	DPL687(13)	7.96	12/11	1.51**	2.79**	0.29	11/22	11/11
BNL243(18)	CER165(13)	7.94	12/22	0.76*	0.29	0.99*	22/11	12/11
DPL564(6)	TMB1296(5)	6.16	12/22	1.64**	1.31*	0.97	22/11	22/22
GH256(11)	HAU1371(6)	9.67	12/11	0.43	1.69**	0.40	22/22	12/22
BNL2960(10)	CIR307(1)	7.36	12/11	2.63**	2.92	1.78**	12/11	11/11
NAU3519(12)	JESP056(2)	5.97	12/11	-0.47	0.19	0.57	22/22	22/11
BNL3261(12)	CGR6695(2)	5.09	12/11	-1.37*	-0.20	-0.67	22/22	12/22

See footnotes of Table 7 for explanations

**Table 9:** The best and the worst double heterozygotes in the two loci combinations consistently showing significant DD interaction for bolls/plant in F_2: 3_ population.

Locus 1^a^	Locus 2^a^	Var%	Genotype^b^	Over midparent^c^	Over GX1135^d^	Best genotype^f^	Worst genotype^g^
BNL3447(5)	GH137(16)	5.53	12/12	-0.73*	-0.34	22/11	12/11
C2_115(12)	DC40250(21)	6.69	12/12	1.54**	0.90*	11/12	22/22
CER165(13)	NAU3839(14)	4.99	12/12	0.54	0.32	11/22	22/22
CGR115(12)	DC40250(21)	6.69	12/12	1.54**	0.90*	11/12	22/22
CGR6864(25)	GH631(12)	4.65	12/12	2.40**	1.97**	11/12	22/22
DPL551(24)	DPL687(13)	5.67	12/12	-0.32	-1.26**	22/12	22/11
BNL3261(12)	CGR6695(2)	4.46	12/12	-0.70*	0.48	22/22	12/22

See footnotes of Table 7 for explanations

## Discussion

### Necessity for Constructing a High-Density Linkage Map in Upland Cotton

Microsatellite markers were widely used, even as framework markers, to construct linkage maps in crops [72], because of their relatively high polymorphisms, detectability, and stability in genome. Although the available EST-SSR sequences are now rich in GenBank, these sequences are so conserved that the polymorphism of SSR markers developed is comparatively low in Upland cotton [15].

Since the first genetic linkage map was constructed in an F_2_ population derived from crosses between *G*. *hirsutum* and *G*. *barbadense* [5], a variety of linkage maps have been constructed and a number of QTLs for yield and fiber quality traits have been identified from these linkage maps. However, heterosis of yield and yield components had not been analyzed in these high-density maps.

In order to construct a high-density linkage map for analysis of yield heterosis in Upland cotton, 450 polymorphic primers were used to construct a linkage map in 173 F_2: 3_ progeny lines derived from two mapping parents ‘GX1135’ and ‘GX100-2’. The genetic linkage map was constructed with 421 loci mapped and coverage of 3814 cM genetic length in the cotton genome. In this research, the majority of primers with high rate of polymorphism were developed by genomic clones from microsatellite-enriched cotton DNA libraries which were sequenced to identify SSR-containing target regions and SSR-containing EST collections [73] (S1 Table). Physical map of D_5_-genome cotton species, A_2_-genome cotton species, and AD_1_-genome in Upland cotton have been completed [21–25]. The physical map of whole genome in Sea Island cotton is not currently available, but the sequencing of this physical map will be released soon (www.cottongen.org). These physical maps will provide extensive information in constructing high-density genetic linkage map, molecular marker selection, and map-based cloning in Upland cotton. With emergence of next generation sequencing technology and more sequences from newly released expression DNA libraries, more and more functional markers will be developed for gene mapping and tagging.

### Genetic Effects of QTL for Yield and Yield Components

In present study, only a few QTLs showed overdominance while majority of them showed partial dominance. Alleles from both female and male parents were in direction of increasing phenotypic variation, which has been observed in previous studies [39–41].

There were a number of QTLs for yield traits that were detected simultanuously in F_2: 3_ and F_2: 4_ populations with both additive and dominant types of genetic effects. QTLs identified for yield and yield components simultaneously in F_2: 3_ and F_2: 4_ populations as shown in Table 3 were further analyzed. Generally, QTLs detected in F_2: 3_ generation had larger effects than those in F_2:4_ generation which indicated depression. For their QTL effects, the dominance effect of *qSY-chr14-1* was 3.32 in F_2: 3_ generation, larger than its dominance effect, 0.10, in F_2: 4_ generation. For lint percent, seven QTLs were detected in both populations with similar magnitudes of additive and dominance effects in two populations. So the effects of the QTLs detected in F_2: 3_ population were substantially larger than those detected in F_2: 4_ population. Same phenomenon was observed in a previous study of QTLs for yield using F_2_ and F_2: 3_ populations using a vegetatively reproduced rice [74]. These results are consistent with the contention that dominance effects were the genetic basis of heterosis and inbreeding depression in Upland cotton.

### Repeatability of QTLs among Different Studies

Although a number of QTLs for yield and yield components were identified in earlier reports using both inter-specific populations and intra-specific Upland cotton populations, only a limited number of markers were reported to be common among different populations in cotton genome. Therefore, it is necessary to fill this gap by identification of enough QTLs for yield traits consistent in different segregating populations and different environments. Some QTLs contributing to yield traits were identified on the same chromosomes in different studies. For example, QTLs for lint yield were detected on chromosome 1, 2, 3, 5, 6, 9, 12, 13, 14, 15, 16, 20, 23, 24, 25 and 26 [7, 75–80]. Among these, a common QTL for lint yield on chromosome 14 was detected in different mapping populations and environments [7, 77]. In current study, the QTL *qLY-chr13-1* was also detected on chromosome 13 with interval between markers of GH157 and BNL 1495. Another QTL for lint yield was detected in a study by Wu et al. [79] with flanking markers BNL1421 and BNL1495. These two QTLs for lint yield may be overlapped or a common one due to their location proximity in the genome.

### Digenic Interactions in F_2: 3_ and F_2: 4_ Populations

A total of nine genotypes were detected with significant interactions in three types of two-locus epistasis. In all interaction types, AA was an interaction for genotypes homozygous in both parents, including 11/11, 11/22, 22/11 and 22/22 (11 representing Parent 1 and 22 representing Parent 2); AD/DA was interaction for genotypes homozygous in one of parent and heterozygous in another parent, including 11/12, 22/12, 12/11 and 12/22; DD was interaction for genotypes heterozygous in both parents, i.e., 12/12 [41–42].

In F_2: 3_ and F_2: 4_ populations, three types of digenic interactions, AA, AD/DA and DD, were detected. AA interactions were detected with the highest frequency and DD interactions were detected with the lowest frequency. None of the double heterozygotes was detected as the best genotypes in terms of bolls per plant. The large number of significant digenic interactions for yield traits identified in F_2: 3_ and F_2: 4_ populations indicated that epistasis was the genetic basis of heterosis in Upland cotton.

In two-locus combinations showing significant AA effects, the best genotypes for bolls per plant were complementary two-locus homozygotes (11/22 or 22/11). Only one single heterozygote (11/12) was detected as the best genotype. The high heterotic values of two-locus combinations with significant AD/DA interactions indicated that the complementary two-locus homozygotes (11/22 or 22/11) were the best genotypes. In two-locus combinations with significant DD, the best genotypes were frequently single heterozygotes (11/12 and 22/12). No double heterozygote (12/12) was detected as the genotype with best performance. Similar results were also reported in previous studies by Hua et al. [41] and Zhou et al. [44]. These phenomena indicated a low correlation between heterozygosity of parental genotypes and their performance. However, single locus heterozygotes showed higher correlations between parental genotypes and their performance than those of double heterozygotes. These results implied that the double heterozygous does not necessarily favor the expression of a trait. The lack of correlation between heterozygosity and performance is possibly due to the small portion of heterozygous loci analyzed for heterosis [41]. The lack of correlation could also be caused by low association between marker heterozygosity and QTL [81].

Genetic basis of heterosis has been analyzed extensively by different research groups [37–41, 44, 82]. Due to different materials and methods used, these reported genetic and molecular mechanisms underlying heterosis were inconsistent in previous studies. In this research, we dissected the genetic basis of heterosis in F_2: 3_ and F_2: 4_ populations at single- and two-locus levels and concluded that dominance effects at single-locus and epistasis effects at two-locus level were the genetic basis of heterosis in Upland cotton.

## Supporting Information

S1 Fig Variation of lint yield and yield components in F_2: 3_ and F_2: 4_ populations. (a). vertical axis: Number of lines, (b). horizontal axis: Traits values, (c). red arrows: GX1135, blue arrows: GX100-2, black arrows: F1.
(TIFF)

S2 Fig Locations of QTLs for yield and yield component traits in four environments. SY, seed cotton yield; LY, lint yield; BNP, bolls/plant; BW, boll weight;; LP, lint percent. Markers underlined were published previously.
(DOC)

S1 File Traits phenotype value. (XLS)

S2 File Molecular marker data. (XLS)

S1 Table Percentages of polymorphism for the markers between two parents of hybrid ‘Xinza No. 1’. (DOC)

## Abbreviations

cM: centi-Morgan
QTL: quantitative trait loci
RIL: recombinant inbred line
SSR: simple sequence repeat
AA: additive × additive
AD or DA: additive × dominance or dominance ×additive
DD: dominance × dominance
