# Supplementary figures and images for: Partial Dominance, Overdominance and Epistasis as the Genetic Basis of Heterosis in Upland Cotton (Gossypium hirsutum L.)
Source: PLoS One. 2015 Nov 30;10(11):e0143548. doi: 10.1371/journal.pone.0143548 (PMC4664285; doi:10.1371/journal.pone.0143548)

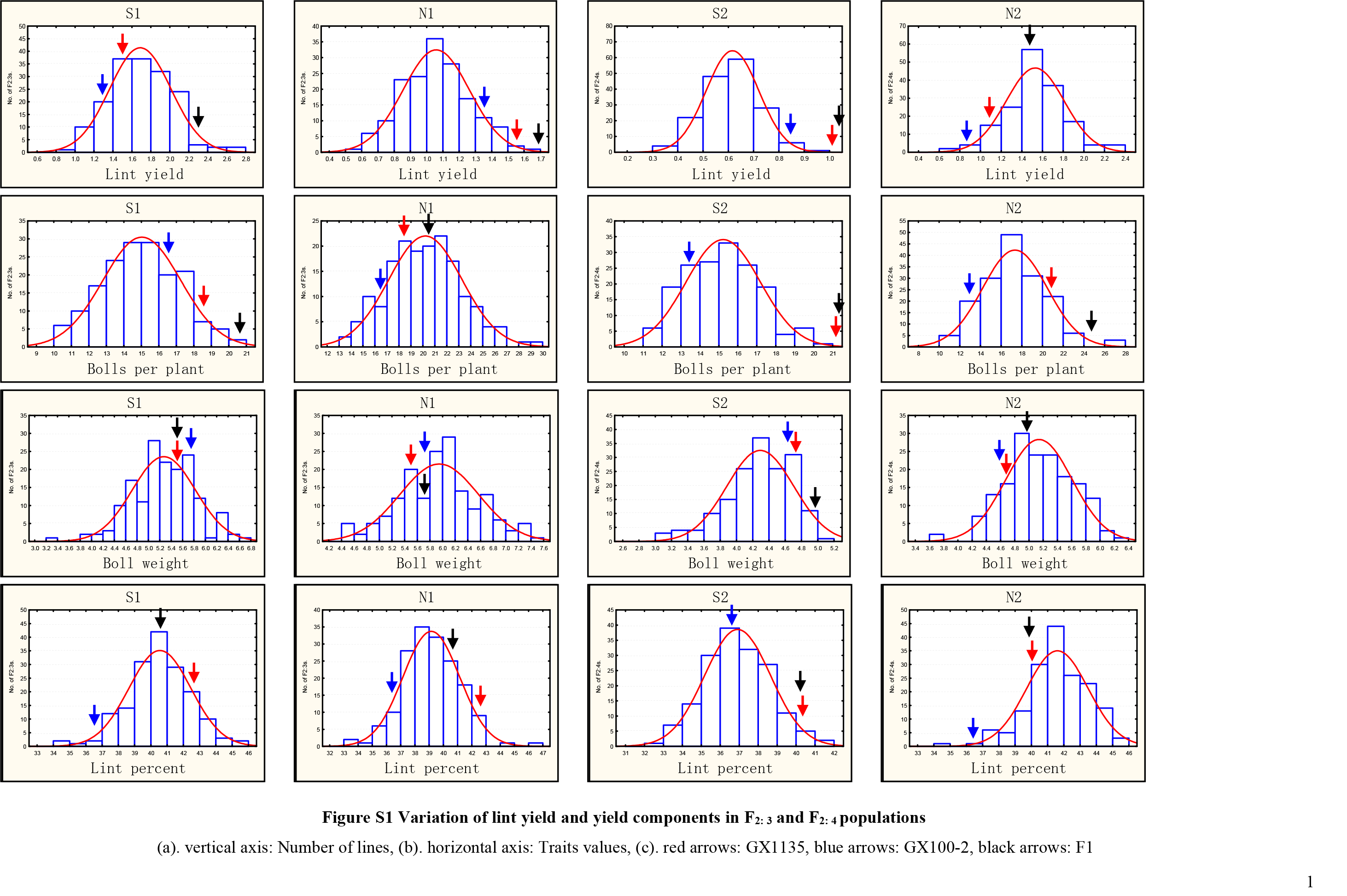

Supplement: S1 Fig — (a). vertical axis: Number of lines, (b). horizontal axis: Traits values, (c). red arrows: GX1135, blue arrows: GX100-2, black arrows: F1. (TIFF) [file pone.0143548.s001.tiff]
