# Supplementary material for: Partial Dominance, Overdominance and Epistasis as the Genetic Basis of Heterosis in Upland Cotton (Gossypium hirsutum L.)
Source: PLoS One. 2015 Nov 30;10(11):e0143548. doi: 10.1371/journal.pone.0143548 (PMC4664285; doi:10.1371/journal.pone.0143548)
Supplement: S2 Fig — SY, seed cotton yield; LY, lint yield; BNP, bolls/plant; BW, boll weight;; LP, lint percent. Markers underlined were published previously. (DOC) [file pone.0143548.s002.doc]

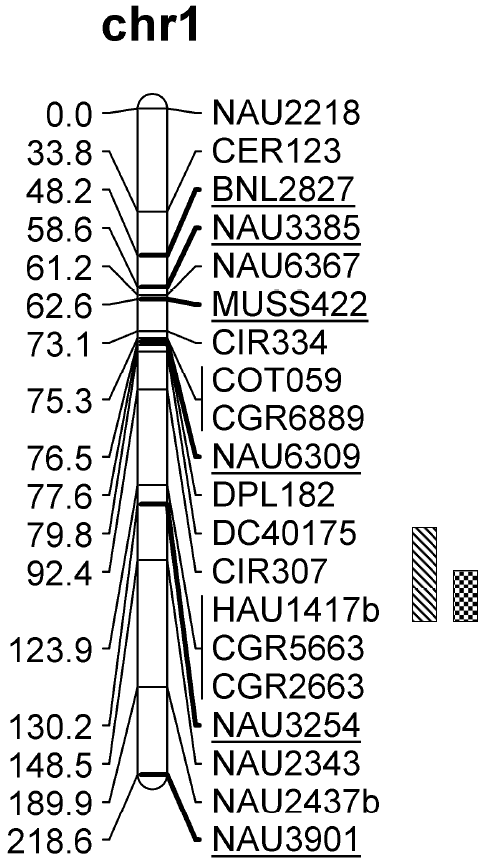

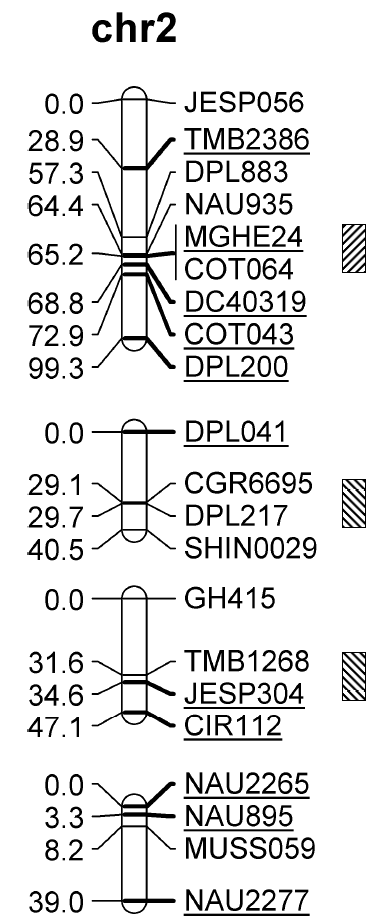

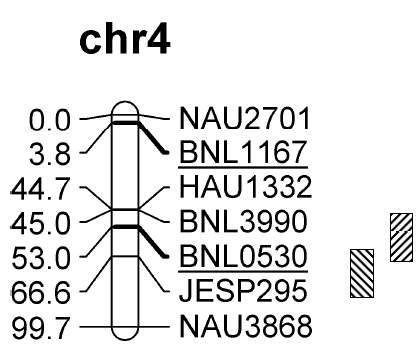

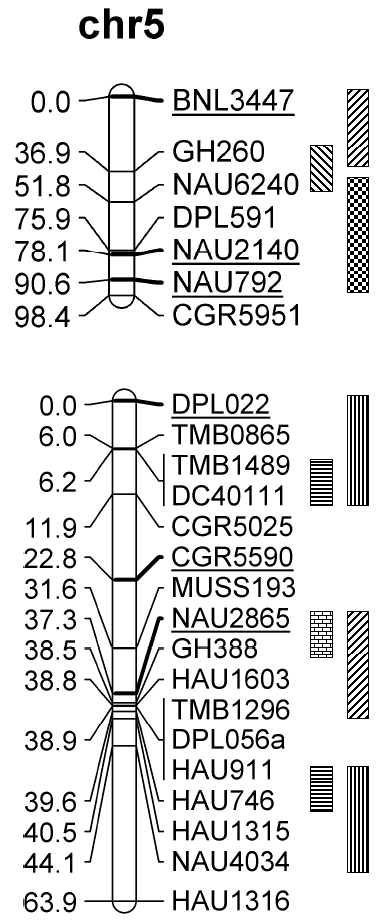

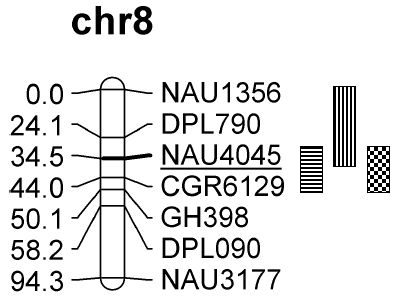

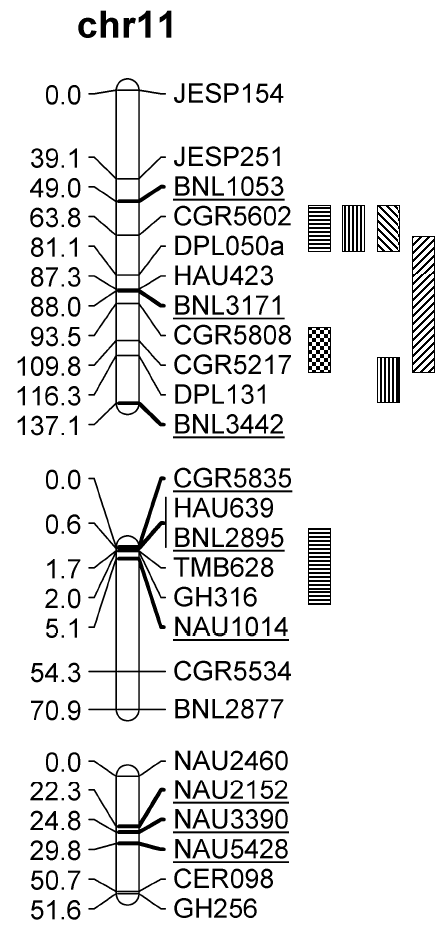

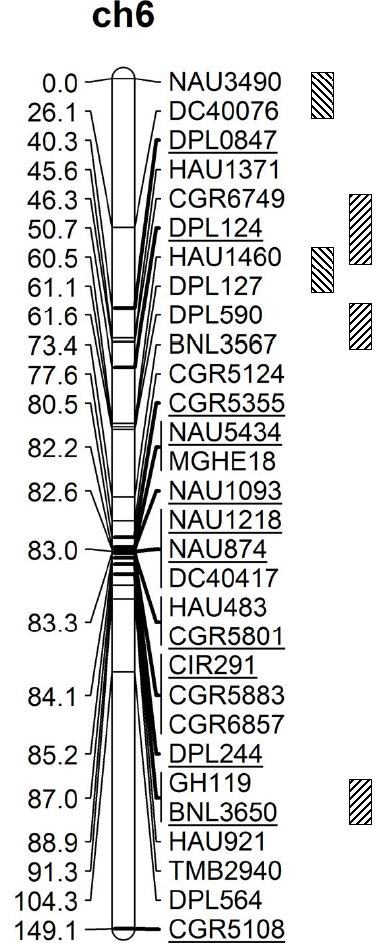

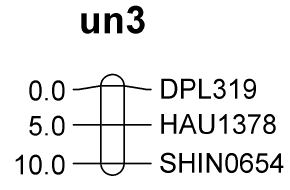

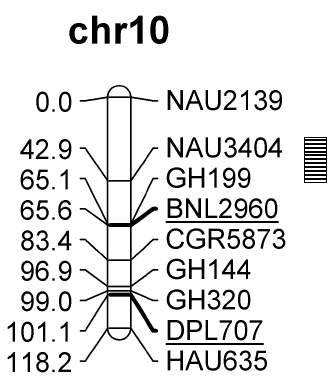

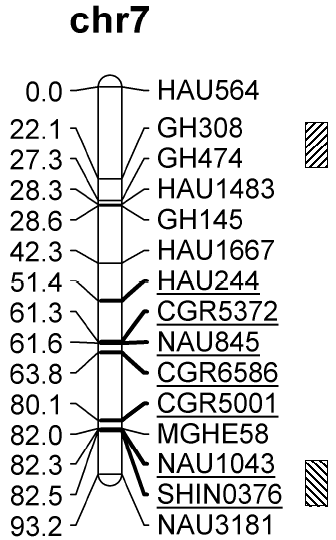

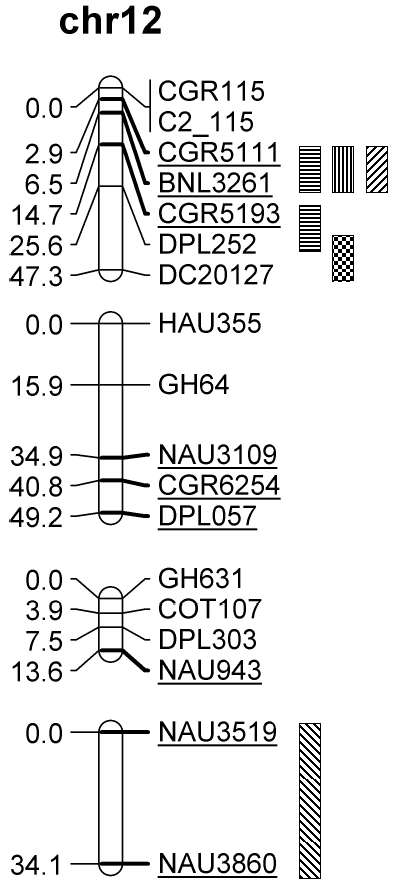

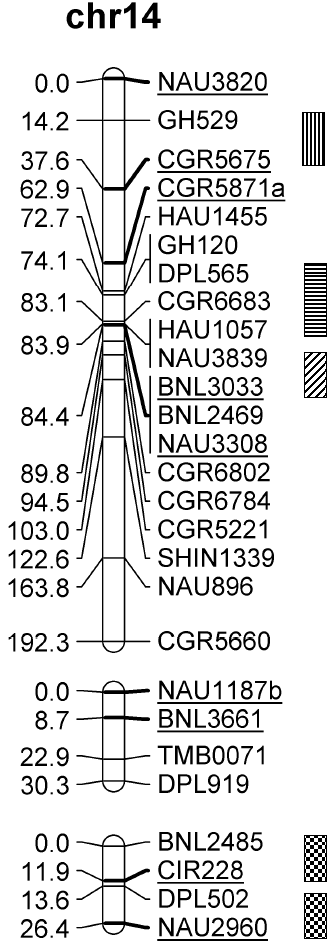

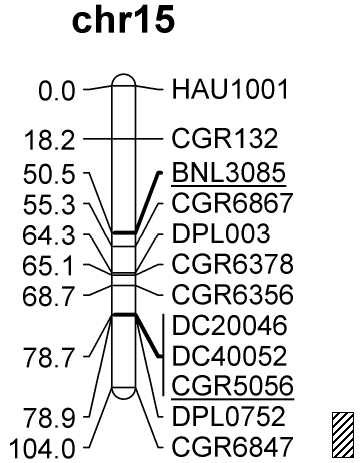

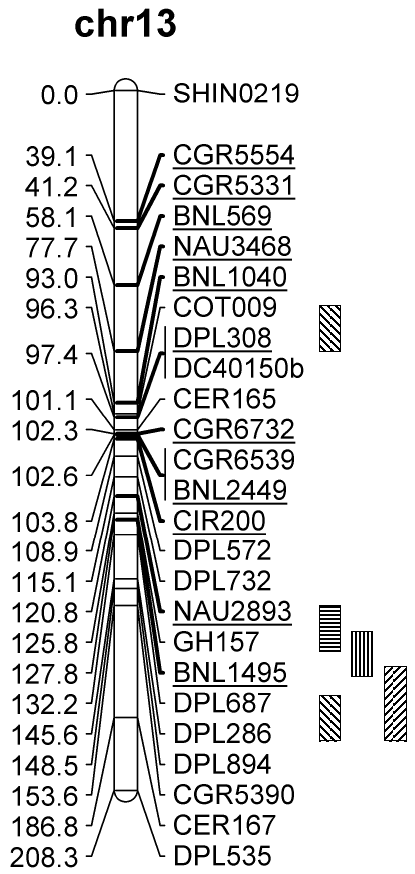

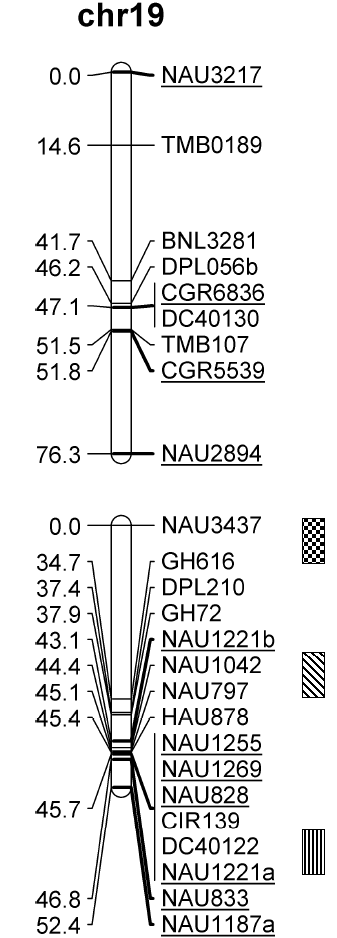

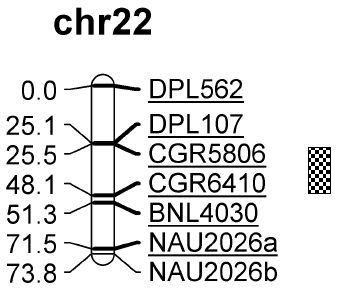

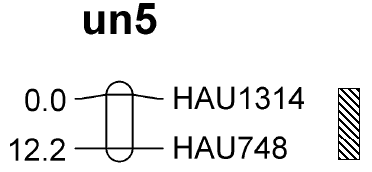

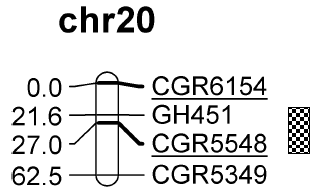

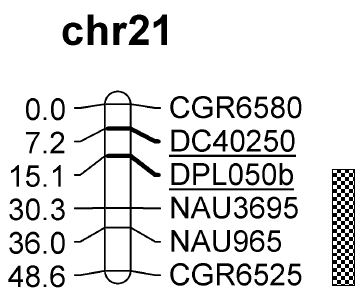

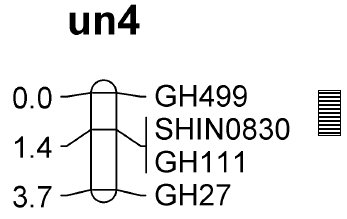

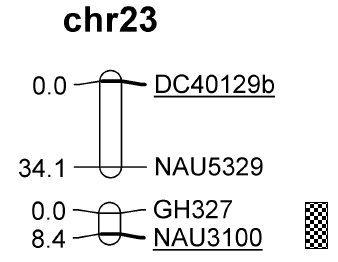

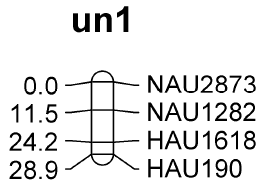

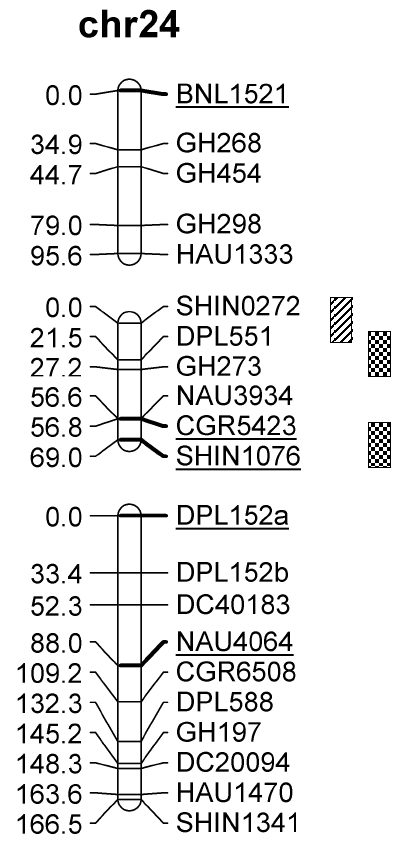

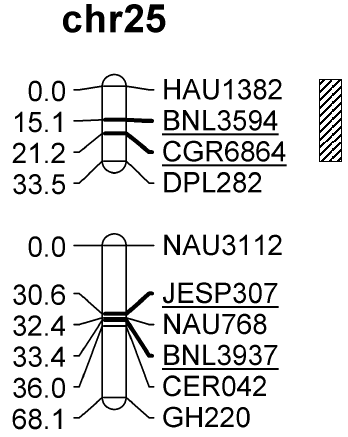

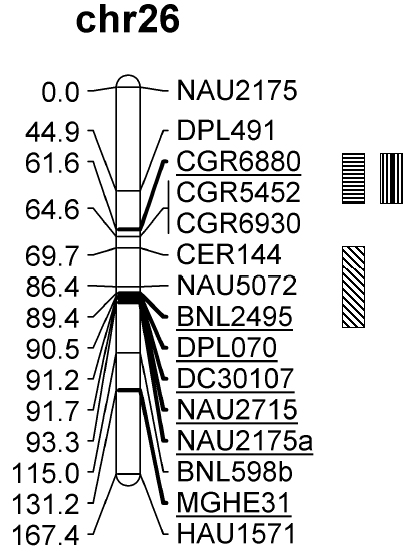

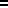

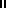

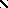

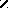

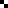


SY LY LP BW BNP

**Figure S2 Locations of QTLs for yield and yield component traits in four environments.**

SY, seed cotton yield; LY, lint yield; BNP, bolls/plant; BW, boll weight; ; LP, lint percent.

Markers underlined were published previously
