# Supplementary material for: Partial Dominance, Overdominance and Epistasis as the Genetic Basis of Heterosis in Upland Cotton (Gossypium hirsutum L.)
Source: PLoS One. 2015 Nov 30;10(11):e0143548. doi: 10.1371/journal.pone.0143548 (PMC4664285; doi:10.1371/journal.pone.0143548)
Supplement: S1 Table — (DOC) [file pone.0143548.s005.doc]

**Table S1** Percentages of polymorphism for the markers between two parents of hybrid ‘Xinza No. 1’

| Marker type | polymorphic markers | Markers number | Polymorphism (%) |
| --- | --- | --- | --- |
| DPL | 54 | 192 | 28.13 |
| MGHES | 7 | 79 | 8.86 |
| CER | 9 | 116 | 7.76 |
| COT | 5 | 67 | 7.46 |
| CGR | 76 | 1194 | 6.37 |
| GH | 38 | 672 | 5.65 |
| BNL | 35 | 661 | 5.30 |
| DC | 20 | 447 | 4.47 |
| SHIN | 12 | 283 | 4.24 |
| C2_ | 3 | 90 | 3.33 |
| CIR | 9 | 376 | 2.39 |
| JESPR | 7 | 297 | 2.36 |
| NAU | 92 | 4798 | 1.92 |
| TMB | 12 | 723 | 1.66 |
| HAU | 39 | 3164 | 1.23 |
| MUSS | 3 | 532 | 0.56 |
